# Application of diffusion kurtosis imaging in neonatal brain development

**DOI:** 10.3389/fped.2023.1112121

**Published:** 2023-03-27

**Authors:** Xueyuan Wang, Xianglong Liu, Meiying Cheng, Desheng Xuan, Xin Zhao, Xiaoan Zhang

**Affiliations:** ^1^ Department of Radiology, Third Affiliated Hospital of Zhengzhou University, Zhengzhou, China; ^2^ Institute of Neuroscience, Zhengzhou University, Zhengzhou, China; ^3^ Department of Radiology, Zhengzhou Central Hospital Affiliated to Zhengzhou University, Zhengzhou, China

**Keywords:** neonate, magnetic resonance imaging, diffusion kurtosis imaging, brain development, mean kurtosis

## Abstract

**Background:**

Deviations from the regular pattern of growth and development could lead to early childhood diseases, suggesting the importance of evaluating early brain development. Through this study, we aimed to explore the changing patterns of white matter and gray matter during neonatal brain development using diffusion kurtosis imaging (DKI).

**Materials and methods:**

In total, 42 full-term neonates (within 28 days of birth) underwent conventional brain magnetic resonance imaging (MRI) and DKI. The DKI metrics (including kurtosis parameters and diffusion parameters) of white matter and deep gray matter were measured. DKI metrics from the different regions of interest (ROIs) were evaluated using the Kruskal–Wallis test and Bonferroni method. Spearman rank correlation analysis of the DKI metrics was conducted, and the age at the time of brain MRI acquisition was calculated. The subjects were divided into three groups according to their age at the time of brain MRI acquisition: the first group, neonates aged ≤7 days; the second group, neonates aged 8–14 days; and the third group, neonates aged 15–28 days. The rate of change in DKI metrics relative to the first group was computed.

**Results:**

The mean kurtosis (MK), axial kurtosis (Ka), radial kurtosis (Kr), and fractional anisotropy (FA) values showed positive correlations, whereas mean diffusion (MD), axial diffusion (Da), and radial diffusion (Dr) values showed negative correlations with the age at the time of brain MRI acquisition. The absolute correlation coefficients between MK values of almost all ROIs (except genu of the corpus callosum and frontal white matter) and the age at the time of brain MRI acquisition were greater than other metrics. The kurtosis parameters and FA values of central white matter were significantly higher than that of peripheral white matter, whereas the MD and Dr values were significantly lower than that of peripheral white matter. The MK value of the posterior limb of the internal capsule was the highest among the white matter areas. The FA value of the splenium of the corpus callosum was significantly higher than that of the other white matter areas. The kurtosis parameters and FA values of globus pallidus and thalamus were significantly higher than those of the caudate nucleus and putamen, whereas the Da and Dr values of globus pallidus and thalamus were significantly lower than those of the caudate nucleus and putamen. The relative change rates of kurtosis parameters and FA values of all ROIs were greater than those of MD, Da, and Dr values. The amplitude of MK values of almost all ROIs (except for the genu of the corpus callosum and central white matter of the centrum semiovale level) was greater than that of other metrics. The relative change rates of the Kr values of most ROIs were greater than those of the Ka value, and the relative change rates of the Dr values of most ROIs were greater than those of the Da value.

**Conclusion:**

DKI parameters showed potential advantages in detecting the changes in brain microstructure during neonatal brain development.

## 1. Introduction

In recent years, evaluation of the developing brain has been attracting increasing attention (1). Deviations from the typical developmental trajectory are supposed to underlie many early childhood diseases (2). Therefore, characterizing the normal development pattern of the early brain is critical. Brain development during infancy is a complex dynamic process, especially during the third trimester and the first month of life, when the brain grows at an extraordinary rate (2, 3). Owing to its high spatial resolution and tissue contrast, magnetic resonance imaging (MRI) is often used to assess brain development to observe structure-specific developmental status (4). In addition, the non-invasiveness and the non-ionizing radiation of MRI render it an ideal procedure for the evaluation of neonates during the early developmental stages, as they are more vulnerable to radiation at that time (5). Brain development of infants is accompanied by a gradual decrease in water content and an increase in the density of macromolecules such as myelin. These changes could be observed as macroscopic T1WI and T2WI signal changes on conventional MR images (6–8). However, the changes observed by the naked eye are subjective and cannot be quantified. Diffusion tensor imaging (DTI) is a diffusion imaging technique that considers water diffusion as a Gaussian process. The main metrics of DTI include apparent diffusion coefficient (ADC) and fractional anisotropy (FA) (9). During brain development and maturation, increased density of cell membranes, interstitial cells, and axons of myelination leads to decreased ADC values and increased FA values in the white matter (WM) (10). However, the complex microstructure in biological tissue makes the water diffusion deviate from the Gaussian distribution, thus reducing the sensitivity and accuracy of DTI (11).

Diffusion kurtosis imaging (DKI) is an extension of DTI, which is based on the principle that water diffusion follows a non-Gaussian distribution, and can be used to evaluate the complex changes in the microenvironment within the tissue structure (12–14). DKI has gradually been applied to research on human brain development. Some studies (10, 15) found that although the DKI metrics changed relatively slowly after the age of two years, the mean kurtosis (MK) value continued to increase compared with the FA value, and the MK developmental plateaus were reached at later ages than the FA value. Gao et al. pointed out that compared with DTI, DKI index showed more specificity for WM microstructure changes between neonates with simple T2 hyperintensity (T2h) and normal controls for both preterm and full-term neonates, and between neonates with simple T2h and complex T2h with hypoxic–ischemic encephalopathy (16). Moreover, the MK value was also more sensitive to the changes of gray matter. No appreciable change was seen in the FA value of the putamen through the first 4 years and 7 months of life, whereas the MK value of the putamen had increased by 81% (15). Ouyang et al. observed decreased cortical MK throughout the study period in 89 preterm neonates aged 31–42 postmenstrual weeks, whereas the decrease in cortical FA reached its plateau around 37 weeks (17). Some studies (17–19) found that the DKI metrics of neonates showed a significant correlation with age, and the development pattern differed in different regions of the brain. Meanwhile, it was also observed that the MK value changed significantly faster than other metrics during brain development (10, 15). Therefore, DKI is a feasible technology in the study of neonatal brain development. However, few studies have analyzed the trend and extent of changes in DKI metrics in different brain regions during the neonatal period. In this study, we aimed to characterize the change patterns of DKI metrics in both WM and gray matter during neonatal brain development.

## 2. Materials and methods

### 2.1. Subjects

A total of 42 full-term neonates (28 male, 14 female) who underwent conventional MR and DK imaging from May 2020 to June 2022 in the Third Affiliated Hospital of Zhengzhou University were enrolled in our study. None of these neonates had central nervous system disease, and they had mainly attended the hospital for the treatment of facial hemangioma, neck hemangioma, oculopathy, sinonasal abnormalities, etc. Preterm infants and neonates who had medical histories with possibly related psychiatric/neurologic diseases were excluded from this study. The brain MRI examinations of all subjects were normal. Jin et al. found that the FA value of neonatal WM increased slightly in the first two weeks of life and its growth accelerated at 15–28 days (20). Moreover, some studies found that MRI performed in the second week after birth for some diseases seemed to have a stronger correlation with the outcomes (21). Therefore, the study subjects were divided into three groups (Table 1) according to their age at the time of brain MRI acquisition, as follows: the first group, age ≤7 days (*n* = 14); the second group, age 8–14 days (*n* = 13); and the third group, age 15–28 days (*n* = 15). This research was approved by the ethics committee of the Third Affiliated Hospital of Zhengzhou University. Written informed consents were obtained from the guardians of all participants before the examination.

**Table 1:** General demographics of neonates.

	Neonatal age (days)			* *
Demographic	First group (≤7)	Second group (8–14)	Third group (15–28)	*P*
Number	14	13	15	
Gender				0.845
Male	10	9	9	
Female	4	4	6	
Mode of delivery				0.849
Natural birth	10	8	9	
Cesarean section	4	5	6	
Gestational age (week)	38.561 ± 1.329	37.857 ± 0.821	38.228 ± 1.292	0.315
Birth weight (g)	3,127.143 ± 315.922	3,106.154 ± 423.842	3,180.667 ± 236.537	0.460
Reason for hospital visit				0.733
Hemangioma	8	7	7	
Oculopathy	0	2	1	
Sinonasal abnormalities	3	3	3	
Trauma factors	0	1	2	
Physical examination	2	0	2	

Fisher's exact text was performed for statistical analysis of gender and mode of delivery, and Kruskal–Wallis test was used for statistical analysis of gestational age and birth weight. The numbers in the columns of gender and mode of delivery represent the number of subjects, and those in the columns of gestational age and birth weight represent the mean value ± standard deviation.

### 2.2. Image acquisition

All neonates were sedated with phenobarbital (5 mg/kg) 30 min before the MRI examination. Conventional T1, T2, T1-FLAIR, T2-FLAIR, DWI, and DKI were performed on a 3.0T MRI imaging scanner (SIGNA™ Pioneer; GE Healthcare, Waukesha, WI, United States). Medical cotton balls were placed in the external auditory canals of the infants for hearing protection, and soundproof sponge pads were placed on both sides of the head for hearing protection as well as prevention of motion artifact. DK images were obtained with the following imaging parameters: directions = 25, TR = 8,200.0 ms, TE = minimum, slice thickness = 4 mm, slice space = 0, field of view = 200 × 200 mm^2^, *b* value = 0, 1,000, 2,000 s/mm^2^. The DKI scan time was 7 min and 23 s.

### 2.3. Data analysis

DKI data were processed using the iQuant software (GE Healthcare, Beijing, China). As the newest AW4.7 version is unable to support the old platform for DKI processing, the GE development team moved the algorithms including the DK image processing from AW4.6 FuncTool platform to the Horos and named it as iQuant. The kurtosis parameters (including MK, axial kurtosis [Ka], radial kurtosis [Kr]) and diffusion parameters (including FA, mean diffusion, axial diffusion [Da], and radial diffusion [Dr]) were calculated by the iQuant software (Supplementary Figure S1).

**Figure 1:**
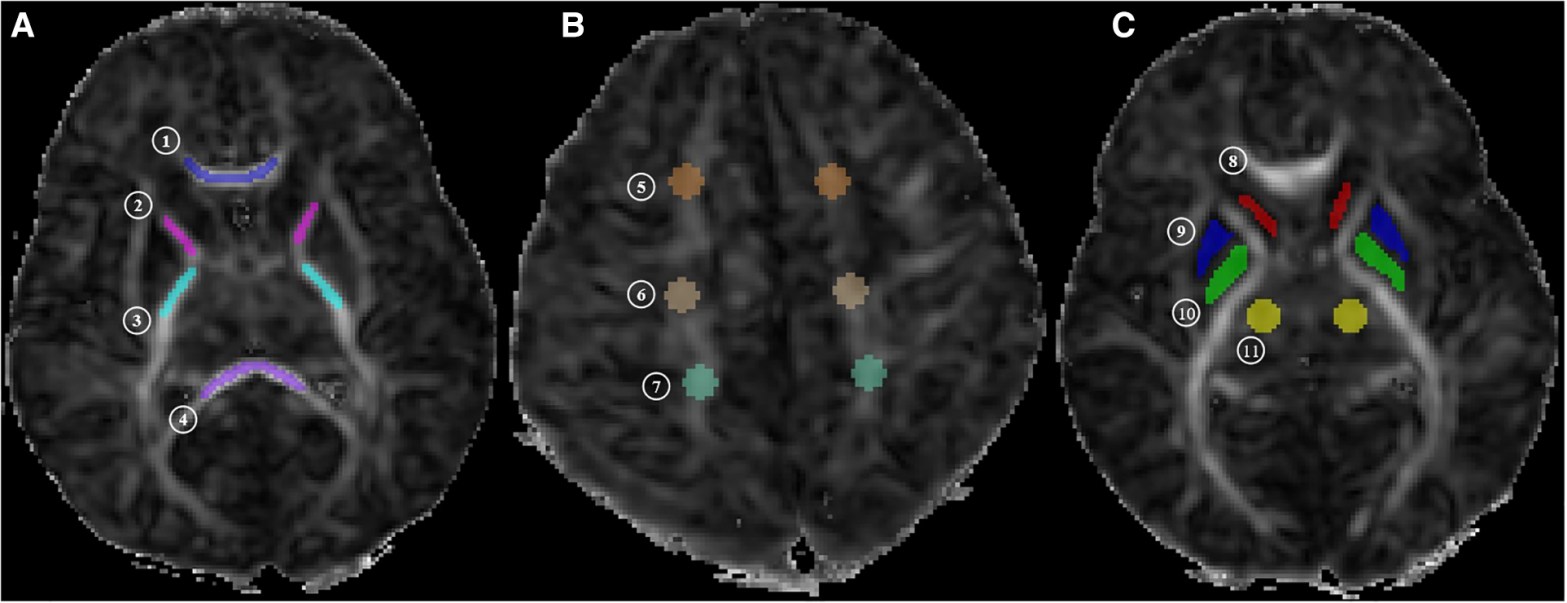
MR image shows. (**A**) Central white matter (level of the basal ganglia: ① genu of the corpus callosum [GCC], ② anterior limbs of internal capsule [ALIC], ③ posterior limbs of internal capsule [PLIC], and ④ splenium of the corpus callosum [SCC]); (**B**) peripheral white matter (level of the centrum semiovale: ⑤ frontal white matter [FWM], ⑥ central white matter [CWM] of the centrum semiovale level, and ⑦ parietal white matter [PWM]); (**C**) gray matter (level of the basal ganglia: ⑧ head of caudate nucleus [CN], ⑨ putamen [PUT], ⑩ globus pallidus [GP], and ⑪ thalamus [TH]). MK, mean kurtosis; Ka, axial kurtosis; Kr, radial kurtosis; FA, fractional anisotropy; MD, mean diffusion; Da, axial diffusion; Dr, radial diffusion.

The regions of interest (ROIs) were drawn by a radiologist with five years of experience and reviewed by a senior radiologist with more than 10 years of experience in brain imaging. The ROIs were drawn on 11 different WM and gray matter structures, including posterior limbs of the internal capsule (PLIC), anterior limbs of the internal capsule (ALIC), splenium of the corpus callosum (SCC), genu of the corpus callosum (GCC), frontal white matter (FWM), central white matter (CWM) of the centrum semiovale level, parietal white matter (PWM), head of the caudate nucleus (CN), globus pallidus (GP), putamen (PUT), and the thalamus (TH) (Figure 1). All ROIs were measured three times by using ITKSNAP (version 3.8.0), and an average value was calculated to minimize error.

### 2.4. Statistical analysis

Data were analyzed using SPSS software (IBM SPSS Statistics 21.0) with a 5% significance level. Spearman rank correlation analysis of the DKI metrics was performed, and the infant age at the time of brain MRI acquisition were calculated. DKI metrics from the different ROIs were interrogated using the Kruskal–Wallis test and Bonferroni method. The DKI metrics of the first group were assumed as the baseline value, and the rate of change in metrics between the different age groups was compared by $P_{\mathrm{change}\;\mathrm{rate}}=(P_{\mathrm{group}}-P_{\mathrm{baseline}})/P_{\mathrm{baseline}}$.

## 3. Results

### 3.1. Images

**Figure 2:**
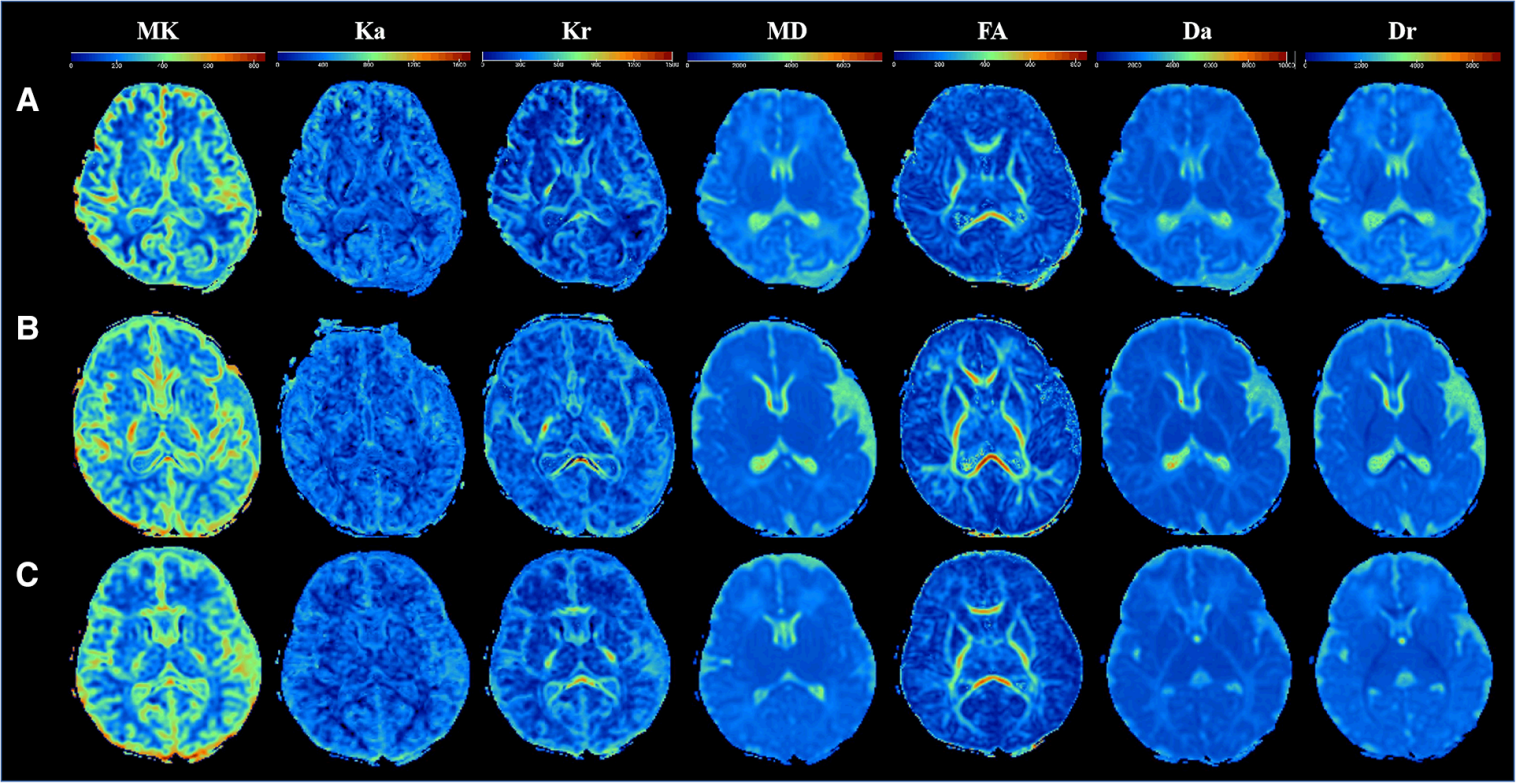
Comparison of DKI multi-parameter images of neonates in different ages. (**A–C**) DKI parameters images of three newborns: (**A**) male, gestational age 37^+5^ weeks, birth weight 2,750 g, weight at examination 2,850 g, cesarean section, age 2 days; (**B**) male, gestational age 37 ^+ 5^ weeks, birth weight 3,100 g, weight at examination 3,800 g, cesarean section, age 14 days; **(C)** female, gestational age 37 weeks, birth weight 3,200 g, weight at examination 4,000 g, cesarean section, age 21 days.

All study subjects had normal brain conventional MR and DKI images. Figure 2 displays the DKI parameter images of full-term neonates. The anatomical structures in the FA, Da, and Dr maps were the most clearly displayed, followed by the MD maps and the kurtosis parameter images. High signal intensity of the posterior limbs of the internal capsule could be observed in the MK and Kr maps. In addition, the signal intensities of the internal capsule and corpus callosum of a 14-day-old neonate and a 22-day-old neonate in the FA map were higher than those of a 2-day-old neonate.

### 3.2. Correlation analysis of DKI metrics and infant age at brain MRI acquisition

As presented in Table 2, there was a significant correlation between all DKI metrics of all ROIs and infant age at the time of brain MRI acquisition (*P *< 0.05). MK, Ka, Kr, and FA values were positively associated with age, whereas MD, Da, and Dr values were negatively associated with age. The correlations between Kr value and age in all ROIs (except CN) were higher than the correlations between Ka value and age. Similarly, the correlations between Dr value and age in all ROIs (except SCC and CWM) were higher than the correlations between Da value and age.

**Table 2:** Correlation between DKI parameters and age at the time of brain MRI acquisition.

ROIs	MK	Ka	Kr	FA	MD	Da	Dr
PLIC	0.923**	0.772**	0.868**	0.911**	−0.718**	−0.690**	−0.762**
ALIC	0.793**	0.705**	0.725**	0.789**	−0.572**	−0.652**	−0.695**
SCC	0.868**	0.749**	0.789**	0.869**	−0.743**	−0.731**	−0.558**
GCC	0.770**	0.685**	0.784**	0.835**	−0.674**	−0.474*	−0.663**
FWM	0.521**	0.469*	0.484*	0.429*	−0.504**	−0.507**	−0.564**
CWM	0.598**	0.566**	0.575**	0.602**	−0.547**	−0.560**	−0.552**
PWM	0.739**	0.584**	0.709**	0.685**	−0.671**	−0.629**	−0.668**
CN	0.621**	0.399*	0.338*	0.413*	−0.605**	−0.407*	−0.422*
GP	0.885**	0.650**	0.661**	0.867**	−0.761**	−0.674**	−0.680**
PUT	0.717**	0.592**	0.627**	0.645**	−0.546**	−0.527**	−0.551**
TH	0.771**	0.632**	0.656**	0.755**	−0.627**	−0.609**	−0.687**

MK, mean kurtosis, Ka, axial kurtosis, Kr, radial kurtosis, FA, fractional anisotropy, MD, mean diffusion, Da, axial diffusion, Dr, radial diffusion, PLIC, posterior limbs of internal capsule, ALIC, anterior limbs of internal capsule, SCC, splenium of the corpus callosum, GCC ,genu of the corpus callosum, FWM, frontal white matter, CWM.central white matter, PWM, parietal white matter, CN, caudate nucleus, PUT, putamen, GP, globus pallidus, TH, thalamus.

Note: The numbers in the table are the correlation coefficients.

– indicates negative correlation; * indicates *P *< 0.05; ** indicates *P *< 0.001.

The correlations between kurtosis parameters and FA values, and age in the CWM were higher than that in the peripheral white matter (PWM). In the CWM, the correlations between kurtosis parameters and FA values, and age were higher than the correlations between MD, Da, and Dr values, and age. The correlations between MK value and age in all ROIs (except GCC) were higher than those between other DKI metrics and age. In the PWM, the correlations between MK value and age in CWM and PWM (except FWM) were higher than those between other DKI metrics and age. The correlation between DKI metrics (except Dr value) and age was PWM > CWM > FWM.

In the gray matter, the correlations between DKI metrics and age in GP and TH were higher than the correlations between DKI metrics and age in CN and PUT. The correlations between MK and FA values and age were higher than those between other metrics and age in GP and TH. The correlations between MK values and age were higher than those between other metrics and age in the CN and PUT.

### 3.3. Differences of DKI metrics between different ROIs

**Figure 3:**
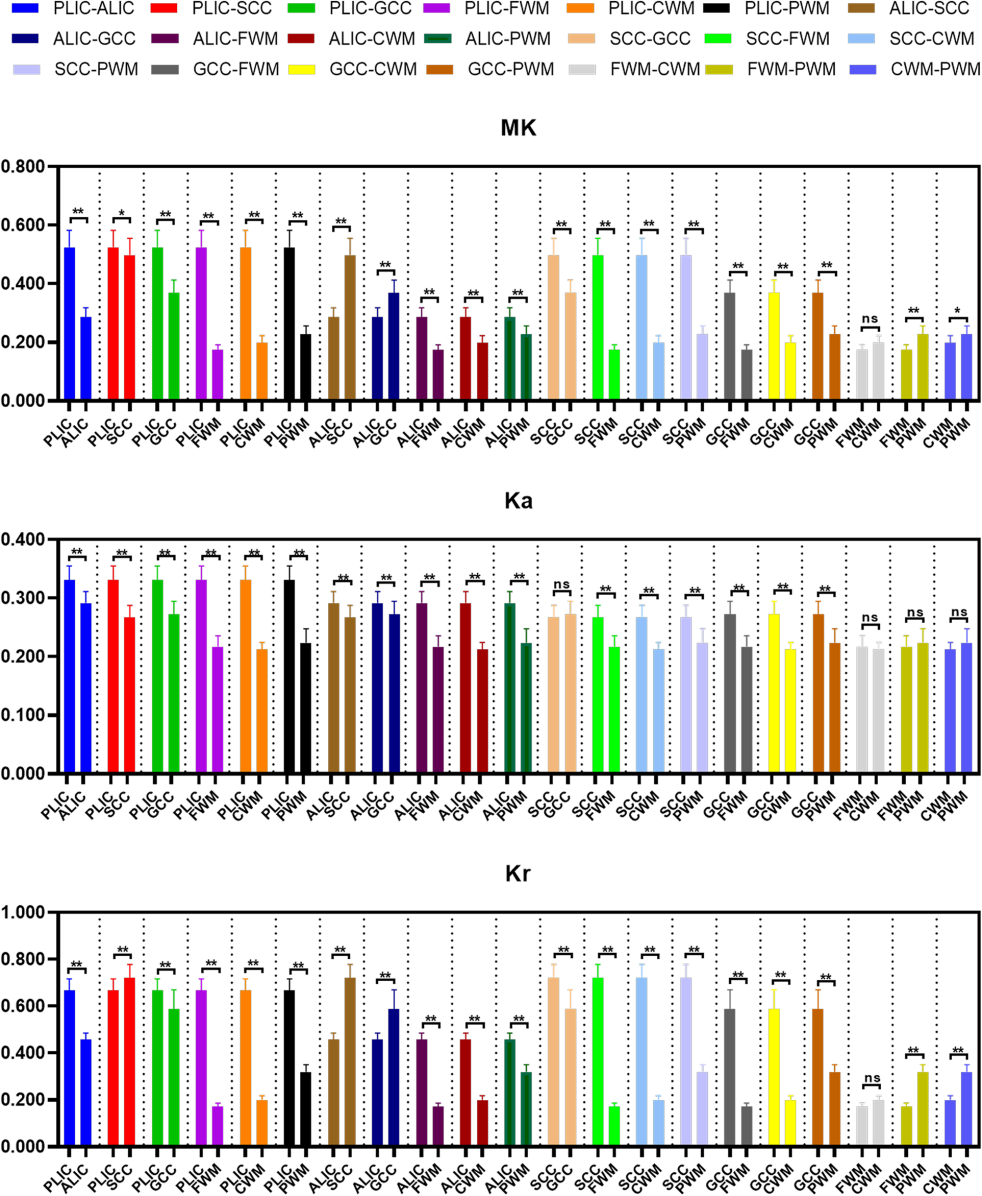
Histograms showing multiple comparisons of kurtosis parameters between different white matter areas. * indicates *P *< 0.05, ** indicates *P *< 0.001, and “ns” indicates no statistically significant difference between the groups.

**Figure 4:**
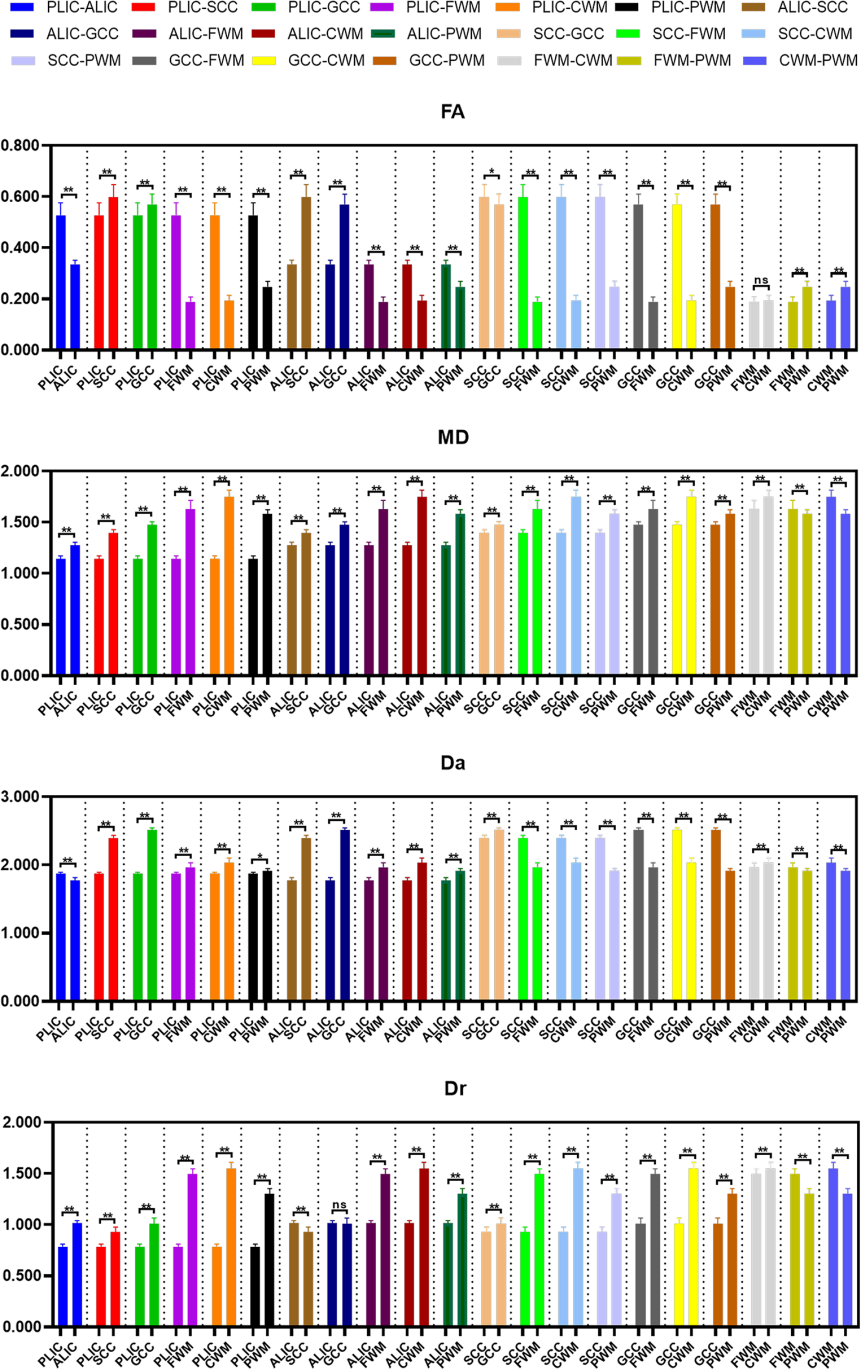
Histograms showing multiple comparisons of diffusion parameters between different white matter areas. The unit of MD, Da, and Dr values is mm^2^/s. * indicates *P *< 0.05, ** indicates *P *< 0.001, and “ns” indicates no statistically significant difference between the groups.

The differences of DKI metrics between different ROIs in the WM were statistically significant (*P *< 0.001). The results of post-hoc test are shown in Figures 3, 4. The MK value of PLIC (0.524 ± 0.058) was the highest among the MK values of the WM. The results of post-hoc test of MK, Ka, and Kr values between most WM ROIs were statistically significant (*P *< 0.05). The MK, Ka, and Kr values of CWM were greater than those of PWM (*P *< 0.001). The results of post-hoc test of Ka values between different WM ROIs were statistically significant (*P *< 0.001). The FA value of SCC (0.598 ± 0.049) was the highest among the FA values of WM. The results of post-hoc test of FA, MD, Da, and Dr values between most WM ROIs were statistically significant (*P *< 0.05). The FA values of CWM were greater than those of PWM (*P *< 0.001). The MD and Dr values of CWM were lower than the MD values of PWM (*P *< 0.001).

**Figure 5:**
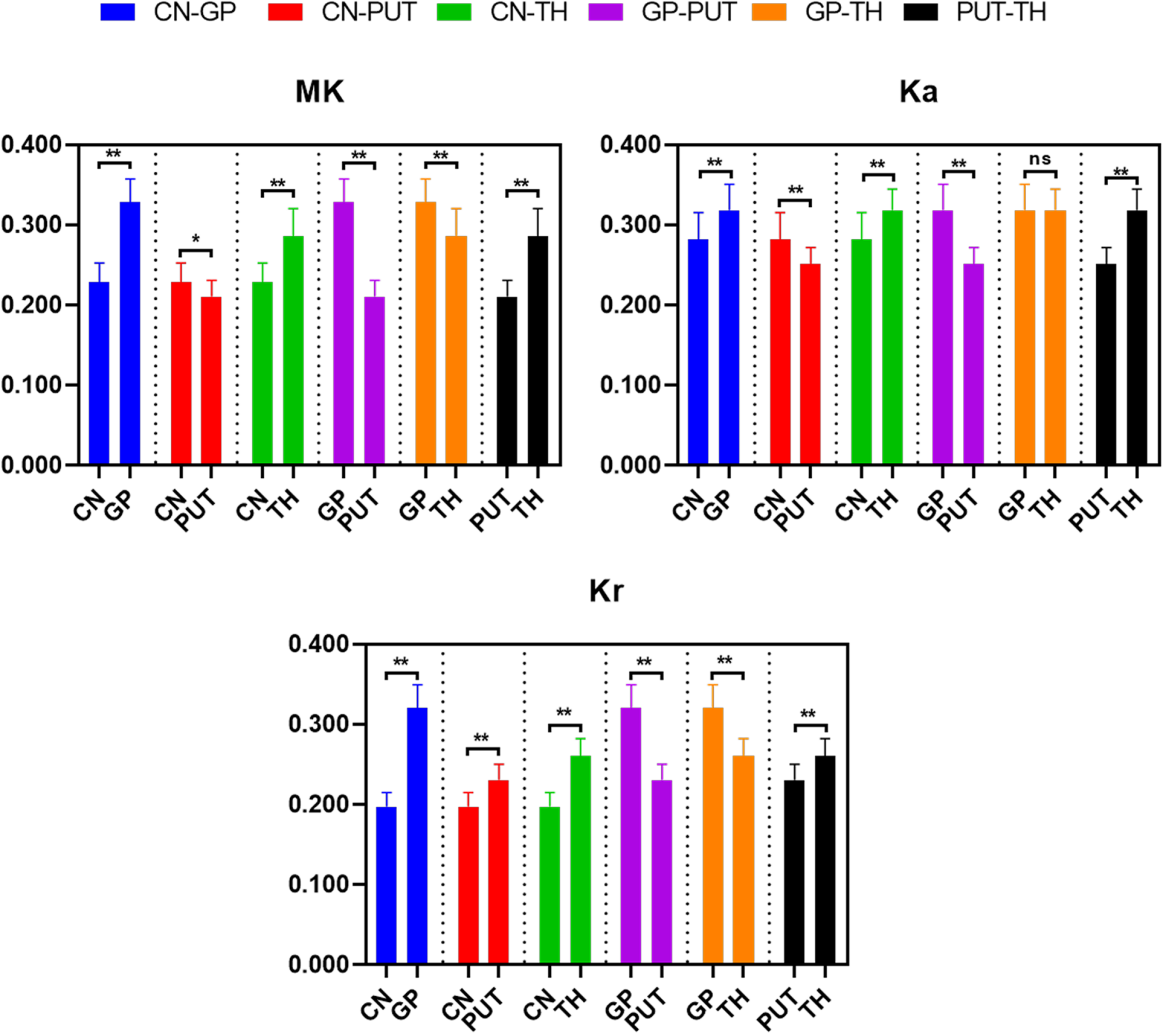
Histograms showing multiple comparisons of kurtosis parameters between different gray matter areas. * indicates *P *< 0.05, ** indicates *P *< 0.001, and “ns” indicates no statistically significant difference between the groups.

**Figure 6:**
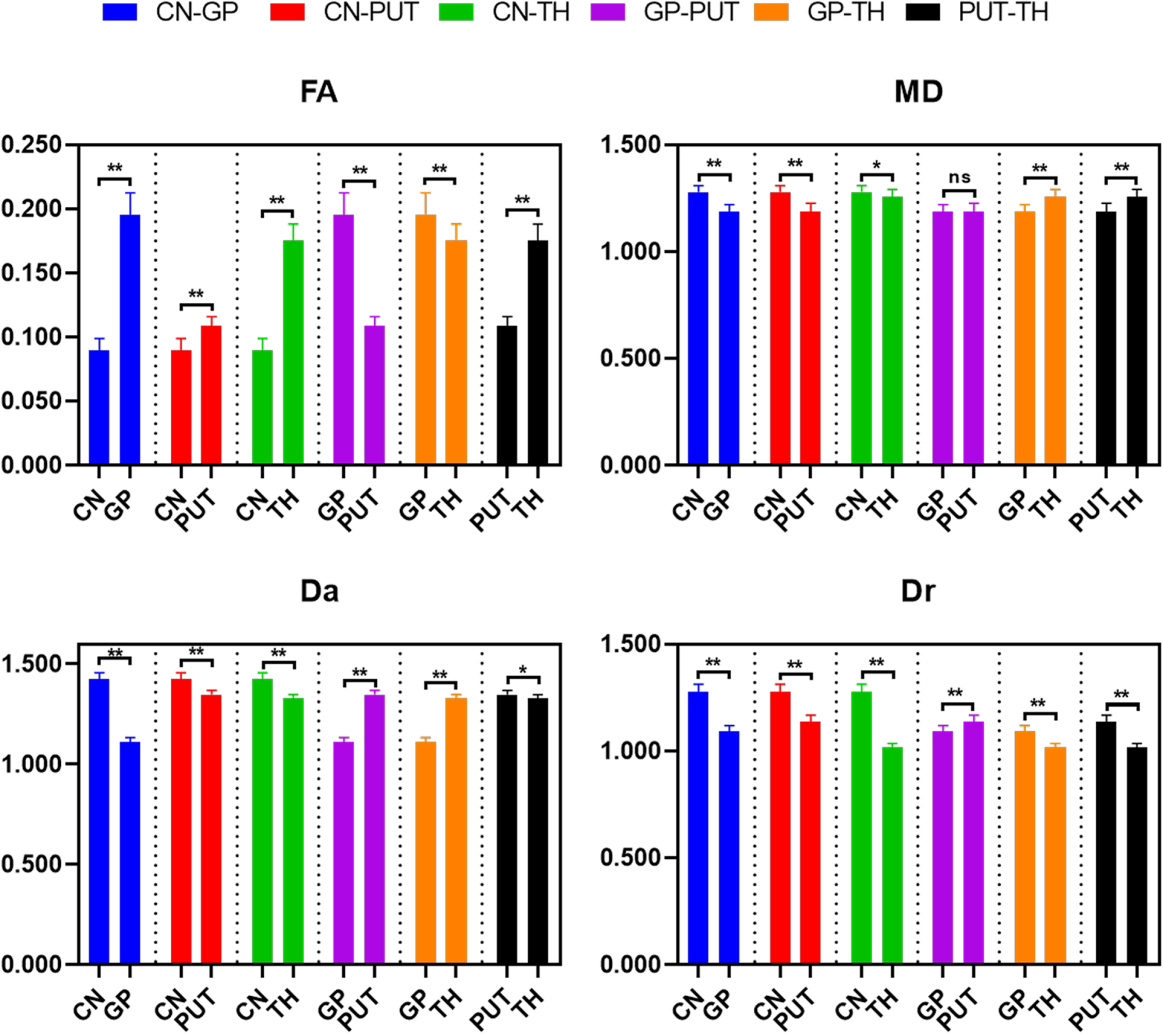
Histograms showing multiple comparisons of diffusion parameters between different gray matter areas. The unit of MD, Da, and Dr values is mm^2^/s. * indicates *P *< 0.05, ** indicates *P *< 0.001, and “ns” indicates no statistically significant difference between the groups.

The differences of DKI metrics between different gray matter ROIs were statistically significant (*P *< 0.001). The results of post-hoc test are shown in Figures 5, 6. The MK and FA value of GP (0.328 ± 0.029 and 0.195 ± 0.017, respectively) were the highest among the MK and FA values of the gray matter. The results of post-hoc test of MK, Ka, Kr, FA, MD, Da, and Dr values between most gray matter ROIs were statistically significant (*P *< 0.05).

### 3.4. Change rates of DKI metrics during the neonatal period

**Figure 7:**
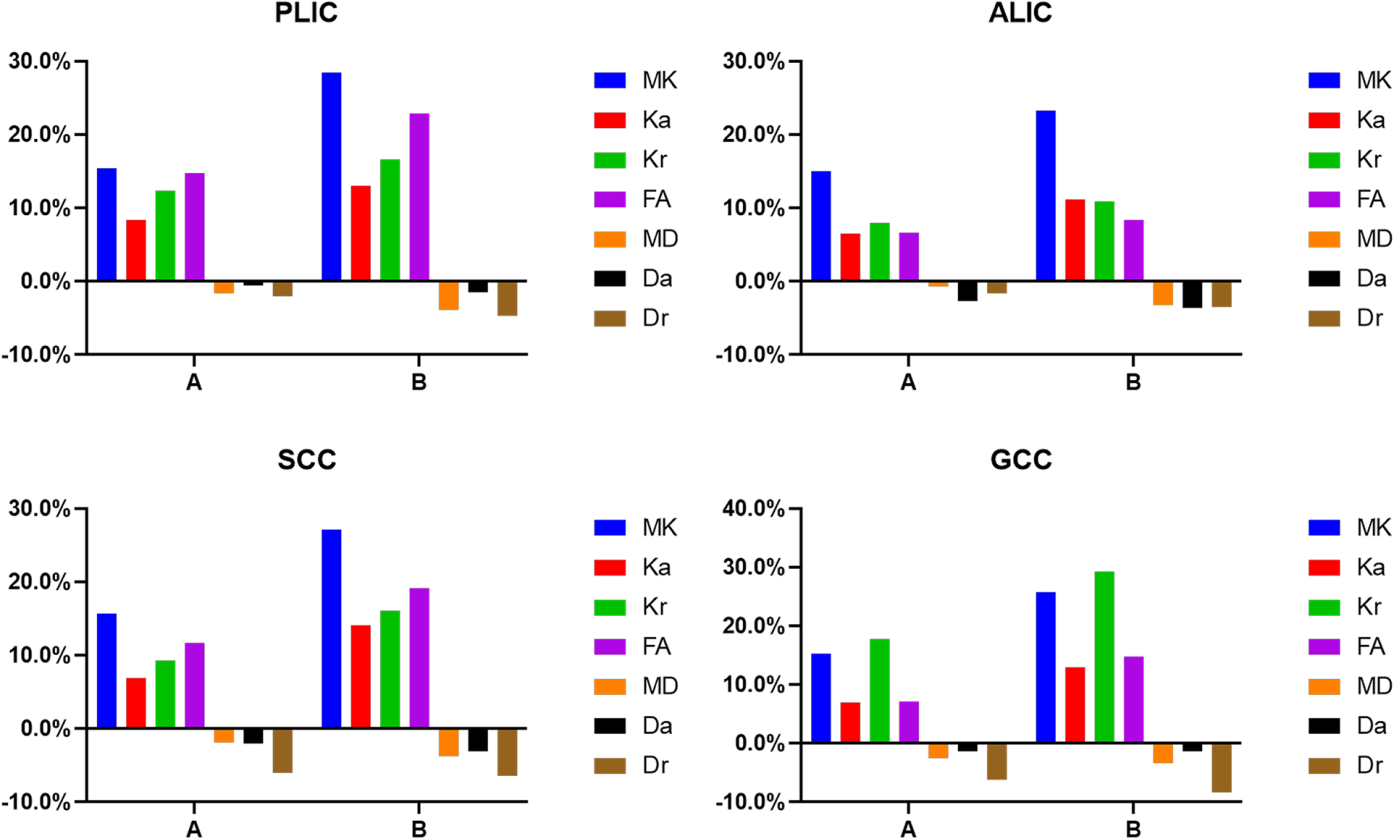
Graphs showing the change rates of DKI parameters of different groups in central white matter. **A** Is the change rate of DKI parameters between the second group (age 8–14 days) and the first group (age ≤7 days). **B** Is the change rate of DKI parameters between the third group (age 15–28 days) and the first group (age ≤7 days).

**Figure 8:**
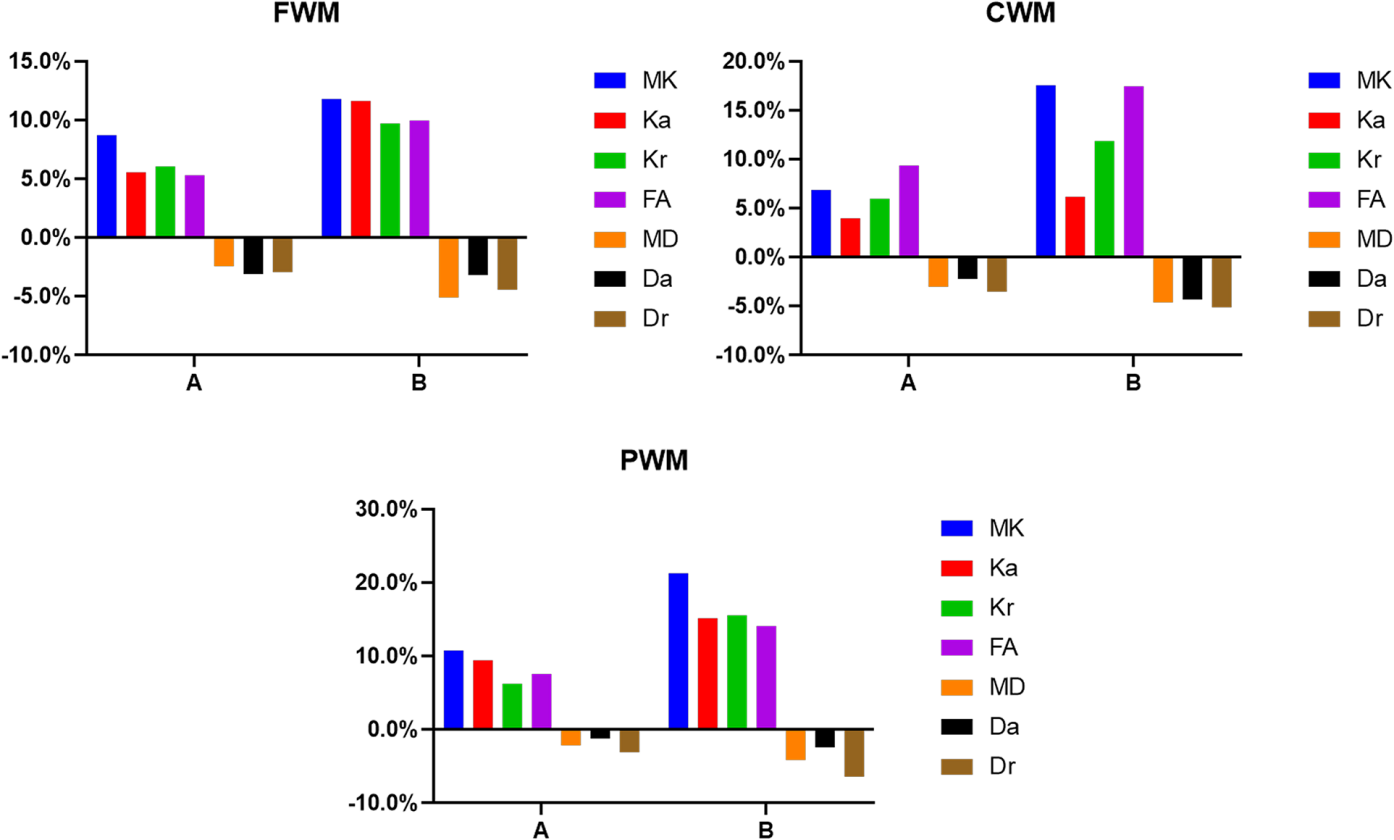
Histograms showing the change rates of DKI parameters of different groups in peripheral white matter. (**A**) Is the change rate of DKI parameters between the second group (age 8–14 days) and the first group (age ≤7 days). (**B**) Is the change rate of DKI parameters between the third group (age 15–28 days) and the first group (age ≤7 days).

**Figure 9:**
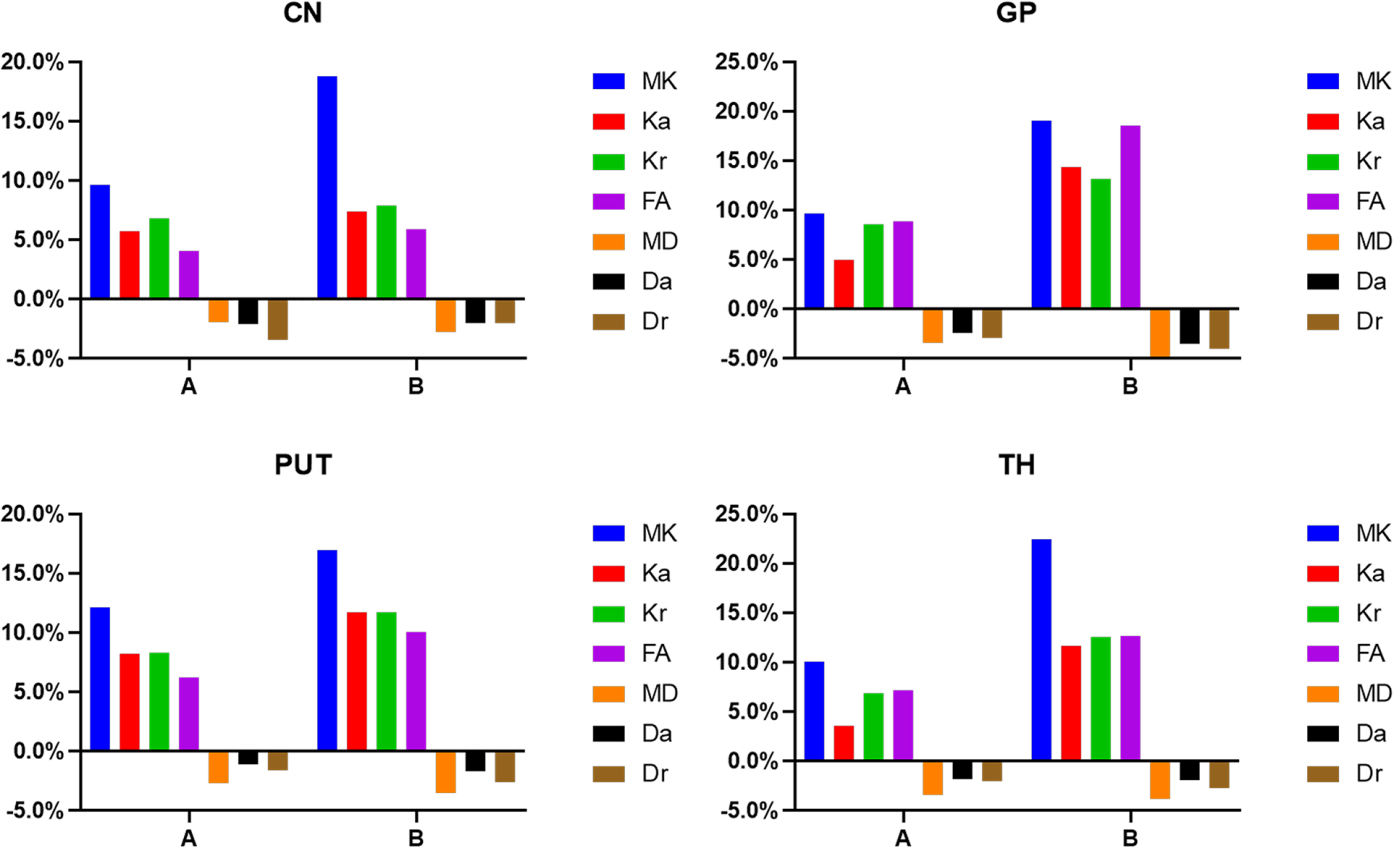
Histograms showing the change rates of DKI parameters of different groups in gray matter. **A** Is the change rate of DKI parameters between the second group (age 8–14 days) and the first group (age ≤7 days). **B** Is the change rate of DKI parameters between the third group (age 15–28 days) and the first group (age ≤7 days).

There were no differences between the three groups in terms of gender, mode of delivery, gestational age, birth weight, and reason for hospital visit (*P *> 0.05; Table 1). The change rates of DKI metrics in WM and gray matter are shown in Figures 7–9 and Supplementary Table S1. The change rates of MK, Ka (except the FWM of group A), Kr, and FA values increased with age, whereas those of MD (except the TH), Da, and Dr values decreased with age. The relative change rates of kurtosis parameters and FA values of CWM, PWM, and gray matter were greater than the relative change rates of MD, Da, and Dr values of CWM.

In the WM, as shown in Figures 7, 8 and Supplementary Table S1, the relative change rates of DKI metrics in Group B were significantly higher than those in Group A. The changes of MK values of WM (except GCC and CWM of group A) were the highest among all DKI metrics. In most WM, the relative change rate of Kr value was higher than that of Ka value, and the relative change rate of Dr value was higher than that of Da value.

In the gray matter, as presented in Figure 9 and Supplementary Table S1, the relative change rates of kurtosis parameter and FA values in Group B were greater than those in Group A, whereas the relative change rates of MD, Da, and Dr values in Groups A and B were similar. In gray matter (except the GP of Group B), the relative change rate of Kr value was higher than that of Ka value, and the relative change rate of Dr value was higher than that of Da value.

## 4. Discussion

With the continuous development of the early brain, WM myelination, synapse formation, and neuronal migration gradually complicate the brain structure; reduce the degree of water diffusion; and increase the degree of anisotropy during the neonatal period. DTI has been widely used to quantify these changes in the pediatric population (5). DKI is a higher-order description of water diffusion on the basis of DTI, and it is better than DTI in detecting the heterogeneity of water diffusion in gray matter and the multiple crossing fibers in WM (22). Our findings show that the degree of increase or decrease of DKI metrics varies in different regions and ages, and the change of MK value is the most significant among all metrics.

Paydar et al. found that the MK and FA values of WM increased nonlinearly with age, and that the changes of parameters were the most obvious in the first two years of life (15). Shi et al. extended the age range of the subjects in their study to 14 years and found that FA, MK, Kr, and Ka values increased with age, whereas MD and Dr values decreased with age. They also found that DKI parameters changed rapidly within the first two years (10). According to previous studies, the DKI parameters changed very quickly within the first two years of life. In our research, only 42 neonates with age ≤28 days were included. Therefore, only the correlation between DKI metrics and age at the time of brain MRI acquisition was analyzed, and whether the correlation was linear was not specifically analyzed. Increasing daily age is accompanied by changes in the degree of myelination, arrangements of the WM fibers, size of the extracellular space, water content, and gray matter structure. In this study, we also found that MK, Ka, Kr, and FA values were positively associated with age whereas MD, Da and Dr values were negatively associated with age. Li et al. (19) discovered that MK and Kr values were highly sensitive to the structural changes of some WM regions (such as corpus callosum) in neonates. Meanwhile, MK values provided independent information from Ka and Kr values, and further improved the performance of Mahalanobis distance in studying the maturational processes in WM. These results illustrated that kurtosis parameters could provide additional information to study brain development. We also found similar results in our research. The correlation between the kurtosis parameters and FA values, and age was higher than the correlation between the MD, Da, and Dr values, and age in CWM. In addition, we found that the DKI metrics of gray matter changed with age. The correlation between the MK values of gray matter and age was the highest among all DKI metrics. This might be attributable to the difficulty in detecting the isotropic changes of gray matter with diffusion parameters. The kurtosis parameters can quantify the diffusion displacement of non-normally distributed water molecules, and detect the degree and heterogeneity of restricted diffusion of water, thereby reflecting the changes of the microstructure more comprehensively.

Cerebral myelination in neonates follows a regular pattern from inferior to superior, back to front, central to peripheral, and projection fibers faster than association fibers (23). When the DKI metrics between different ROIs in the WM were compared, the MK value of PLIC was found to be higher than that of ALIC, SCC, and GCC, and the MK values of CWM were significantly higher than those of PWM. These results were consistent with the normal development pattern of the neonatal brain. Moreover, some studies have shown that the MK value was greatly influenced by the degree of myelination (18). The FA values of SCC and GCC were the highest among the FA values of WM. This might be attributable to the dense fiber packing and anisotropic structure in the corpus callosum, and to the fact that the FA value was more sensitive to the dense fibers (6, 24). Furthermore, some studies have reported that increased FA values were also observed in the unmyelinated regions (19). Sensory and visual functions are very important in all animals. The corpus callosum is an important structure that manage these functions, and the fibers connecting the corresponding areas in the corpus callosum also develop relatively early. In addition, we found that the changes of MK values in WM (except GCC and CWM) were the most significant among all DKI metrics. Only 5% relative change rate of MD values was observed in WM. Some studies have shown that MD values were affected not only by the anatomical structure but also by factors such as macromolecule concentration, whereas MK values were not affected by these factors and had higher specificity (25). Therefore, MK value was more sensitive than MD value in reflecting the microstructural changes in WM. Pre-myelination and myelination in the neonatal period led to the rapid decreases in Da and Dr values, which offset the increase in Da value caused by the increase of fiber number, while the Dr value was not affected (3, 26, 27). The change of Ka value was more pronounced, which more sensitively reflected the axial diffusion limit. The increases in myelin sheath thickness and the axon diameter hindered water diffusion, which resulted in a decrease in Dr value and an increase in Kr value. Our results showed that the change of Kr value was greater than that of Dr value. The change rates of Kr values were significantly greater than those of Ka values in most WM, and the correlations between Kr values and age were higher than the correlations between Ka values and age. These results were also observed in Dr and Da values, owing to the fact that the degree of radial diffusion limitation in WM fibers was greater than that of axial diffusion limitation during the development process. Moreover, MK value omitted the directivity of diffusion, while the Kr and Ka value could complement the deficiency of MK value and improve the directional information of DKI. Jin et al. studied the change of FA value of WM in 133 neonates and found that FA values increased slightly during Days 1–14 and growth accelerated between Days 15–28 (20). However, in our study, the relative change rate of DKI parameters during Days 15–28 was about twice that observed during Days 8–14, which was different from the findings of Jin et al. This might be attributable to the differences in factors such as sample size and the age of the subjects between the studies. Typical development is the result of interaction between genetic, epigenetic, and environmental factors (e.g., external stimuli, maternal, nutritional, or medical factors) (3). Therefore, multicenter studies with large sample sizes are warranted to confirm the results of the present study and make them generalizable.

The MK, Ka, Kr, and FA values of gray matter increased with age, whereas the MD, Da, and Dr values decreased with age. This change of DKI metrics in gray matter was related to the decrease of water content and increase of macromolecular concentration. The binding of water and macromolecules reduced the content of free water, while the signal intensity of bound water was small. During the development of neurons and glial cells, the combination of macromolecules with cell membranes and organelles further impeded the water diffusion (28). In addition, the changes of parameters might also be related to the other special structures involved in gray matter development, such as the increase of cell membranes and organelles, transformation of glial cells, increase of dendrites, etc. (15). Some studies showed that DKI was helpful to study the isotropic structures (such as gray matter), whereas DTI parameters were not sensitive to isotropic structural changes (29). Our study also retrieved similar results. The range of the relative change rate of kurtosis parameters (3.5%–22.4%) was greater than that of diffusion parameters (1.1%–18.5%) in gray matter. In the correlation analysis, the correlation between the DKI metrics of GP and TH, and age was higher than the correlation between the DKI metrics of PUT and CN, and age. When the DKI metrics in different gray matter areas were compared, we found that the kurtosis parameters and FA values of GP and TH were significantly higher than those of CN and PUT, whereas the Da and Dr values of GP and TH were significantly lower than those of CN and PUT. We also found that the relative change rate of MK and FA values was higher than that of CN and PUT in GP and TH during Days 15–28. This might be attributable to the fact that GP and TH are gray matter nuclei but contain WM fibers, and the progressive myelination of fibers increases the anisotropy. The microstructure of GP is the most complex among the gray matter and is composed of myelinated multipolar neurons, and the cytoplasm contains more iron and Nissl bodies. CN and PUT have similar structures, in that neither contains a large number of myelinated fibers, and both have a low iron concentration in the cytoplasm. Mukherjee et al.'s study also supported this result. They found that the diffusion of neonatal basal ganglia conformed the isotropic structure and slightly deviated from the isotropy with age. However, this does not mean the anisotropic development of gray matter itself; rather, it may be caused by the maturation of small WM tracts in the basal ganglia (28). Thus, DKI can detect the changes of microstructure and reflect the heterogeneity in cerebral gray matter of neonates.

There were several limitations to our study. First, our sample size was relatively small. We will continue further studies with larger sample sizes. Second, manual placement of the ROIs might have reduced the reliability and reproducibility of the study. However, the ROI method is easier for clinicians to implement and is widely used in clinics. Third, we had to use cross-sectional data in our study owing to the difficulty in obtaining longitudinal data of the same neonates.

## 5. Conclusion

In conclusion, DKI can reflect the changes of WM and gray matter during the brain development process in neonates. We observed that the MK, Ka, Kr, and FA values increased with age, whereas the MD, Da, and Dr values decreased with age. Neonatal brains show extensive changes in the first month of life. The size and degree of changes of DKI metrics in the different regions of neonatal brain varied, reflecting the differences and changes in the development process of different tissue microstructures. The change of MK value in the WM and gray matter was the most significant among all DKI metrics.

## Data Availability

The original contributions presented in the study are included in the article/**Supplementary material**, further inquiries can be directed to the corresponding authors.
