# Supplementary material for: Application of diffusion kurtosis imaging in neonatal brain development
Source: Front Pediatr. 2023 Mar 27;11:1112121. doi: 10.3389/fped.2023.1112121 (PMC10083282; doi:10.3389/fped.2023.1112121)
Supplement: Supplementary file 1 [file Datasheet1.pdf]

Supplementary Material

Supplementary Table 1 The change rates of DKI parameters of different groups

ROIs	Group	MK	Ka	Kr	MD	FA	Da	Dr
PLIC	A	15.4	8.3	12.3	14.7	-1.6	-0.6	-2.0
	B	28.4	13.0	16.6	22.8	-3.9	-1.5	-4.7
ALIC	A	15.0	6.5	7.9	6.6	-0.7	-2.7	-1.6
	B	23.2	11.1	10.9	8.3	-3.3	-3.6	-3.5
SCC	A	15.7	6.8	9.3	11.7	-1.9	-2.1	-6.1
	B	27.1	14.0	16.0	19.1	-3.8	-3.1	-6.5
GCC	A	15.3	6.9	17.8	7.0	-2.5	-1.4	-6.2
	B	25.8	12.9	29.2	14.8	-3.4	-1.4	-8.4
FWM	A	8.7	5.5	6.0	5.3	-2.5	-3.1	-3.0
	B	11.8	11.6	9.7	9.9	-5.1	-3.2	-4.4
CWM	A	6.8	3.9	5.9	9.4	-3.0	-2.2	-3.5
	B	17.5	6.1	11.9	17.4	-4.6	-4.3	-5.1
PWM	A	10.7	9.4	6.2	7.5	-2.2	-1.3	-3.1
	B	21.2	15.1	15.5	14.0	-4.2	-2.5	-6.4
CN	A	9.6	5.7	6.8	4.0	-1.9	-2.1	-3.4
	B	18.8	7.4	7.9	5.9	-2.8	-2.0	-2.0
GP	A	9.7	4.9	8.5	8.8	-3.4	-2.4	-2.9

	B	19.0	14.4	13.2	18.5	-4.9	-3.5	-4.0
PUT	A	12.1	8.2	8.3	6.2	-2.7	-1.1	-1.6
	B	16.9	11.7	11.7	10.0	-3.5	-1.7	-2.6
TH	A	10.0	3.5	6.8	7.2	-3.4	-1.8	-2.0
	B	22.4	11.6	12.5	12.7	-3.8	-1.9	-2.7

Note: The numbers in the table indicate the change rates of DKI parameters of groups. The unit of change rate is %. A is the change rate of DKI parameters between the second group (8-14 days) and the first group (≤7 days). B is the change rate of DKI parameters between the third group (15-28 days) and the first group (≤7 days). – indicates a negative change.

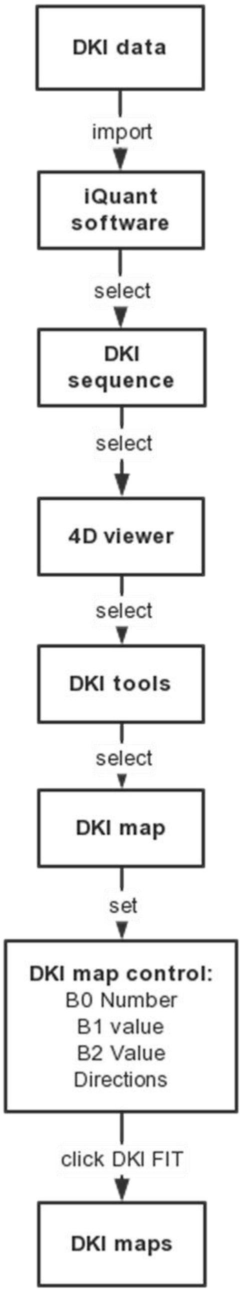

Supplementary Figure 1 Data processing flow. The DKI data is imported into iQuant software, and then the DKI sequence to be processed is selected. Select 4D viewer, then use the DKI map in iQuant tools to set the DKI Map control (including the B0 Number, B1 value, B2 Value, and Directions), and finally click DKI Fit to get the DKI maps.
